# Real-Time Monitoring of Adenosine Triphosphate Fluctuation in Lysosome during Autophagy/Mitophagy

**DOI:** 10.1021/acsami.5c07496

**Published:** 2025-07-11

**Authors:** Jiwen Hu, Hong Wang, Xin Zhang, Chunfei Wang, Anna du Rietz, Mengtao Rong, Caroline Brommesson, Xiongyu Wu, Zhanxiao Wei, Ruilong Zhang, Xuanjun Zhang, Kajsa Uvdal, Zhangjun Hu

**Affiliations:** † Department of Physics, Chemistry, and Biology (IFM), 4566 Linköping University, Linköping SE-581 83, Sweden; ‡ School of Chemistry and Chemical Engineering, and Institute of Physical Science and Information Technology, 12487 Anhui University, Hefei, Anhui 230601, China; § Cancer Centre and Centre of Reproduction, Development and Aging, Faculty of Health Science, 59193 University of Macau, Taipa, Macau SAR 999078, China

**Keywords:** Förster resonance energy transfer (FRET), silica nanoparticles, ratiometric fluorogenic nanoprobe, lysosome targeting, ATP monitoring

## Abstract

Autophagy, a lysosomal degradation pathway critical for cell survival, differentiation, development, and maintaining homeostasis, plays a crucial role in cellular health. Maintaining an adequate level of adenosine triphosphate (ATP), the central molecule in energy metabolism, is equally essential for these processes. However, the interplay between autophagy and energy metabolism remains incompletely understood due to technical challenges, including the need for high-precision, dynamic detection within organelles, and sensitivity to the acidic lysosomal environment. To address these limitations, we developed HR-MP, a ratiometric fluorogenic nanoprobe specifically designed for visualizing ATP levels in acidic lysosomes during autophagy. HR-MP exhibits selective, rapid, and quantitative ATP detection *in vitro*, allowing it to quantitatively monitor lysosomal ATP fluctuations in complex biological environments with excellent biocompatibility, membrane permeability, and lysosome-targeting ability. Importantly, HR-MP enables real-time tracking of ATP fluctuations during starvation- or drug-induced autophagy in living cells, providing a powerful tool for elucidating the links between autophagy and energy metabolism.

## Graphical abstract

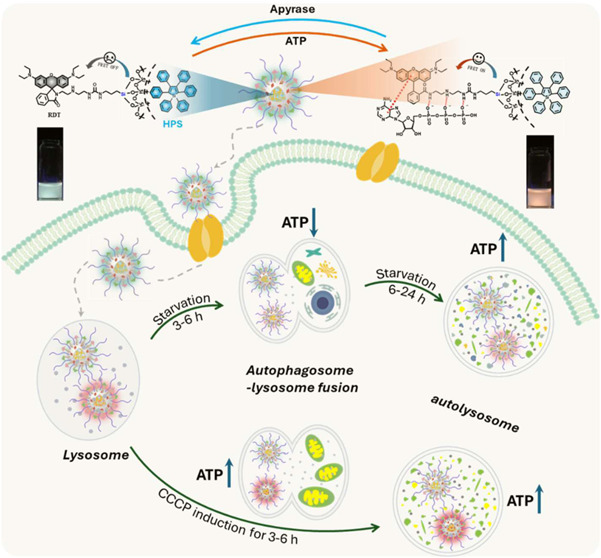

## 1 Introduction

Adenosine triphosphate (ATP) is the primary cellular currency within cells, playing a critical role in maintaining physiological functions and regulating various biological processes, including metabolism, signal transduction, and cell growth.^1^ Mitochondria, as the main site of ATP production, generate approximately 90% of the cell’s ATP through oxidative phosphorylation, thereby providing the energy necessary for cellular activities.^2,3^ Beyond supporting basic cellular functions, as-synthesized ATP in mitochondria also contributes to the regulation of autophagy, a lysosome-mediated degradation pathway that recycles damaged or excess cellular components to maintain homeostasis, especially under stress conditions.^4,5^ Recent studies have shown that ATP levels are closely related not only to energy metabolism but also to various stages of the autophagic process.^6–8^ For instance, the formation of autophagosomes, their fusion with lysosomes, and the functional activity of autolysosomes all depend on sufficient ATP to drive the energy-dependent processes.^9–12^ This underscores that a normal ATP supply is essential for both the initiation and maintenance of autophagy.^8,13^ Furthermore, in response to cellular stressors such as starvation, drug treatment, or oxidative stress, autophagy helps to sustain cellular energy by degrading internal cellular components, thereby preventing cell death.^8^ This highlights a reciprocal relationship: ATP supports autophagy, in turn, contributes to cellular energy balance.^12,14,15^ Interestingly, lysosomes have also been reported to store considerable amounts of ATP, and growing evidence suggests that ATP levels within lysosomes may directly or indirectly influence both lysosomal and mitochondrial functions.^16–18^ In lysosome-mediated mitophagy, ATP fluctuations are closely associated with the functions of these two organelles.^19,20^ Therefore, monitoring ATP dynamics, particularly at the subcellular level, is essential for understanding the regulatory mechanisms involved in mitophagy and the overall intracellular homeostasis. For instance, Li et al. demonstrated that ATP deficiency could induce autophagy in HepG2 cells.^21^ However, real-time quantitative monitoring of ATP levels in lysosomes during autophagy remains challenging, largely because traditional biochemical assays or point-of-care test platforms cannot reveal subcellular distributions of ATP, and genetically encoded sensors are difficult to target precisely to lysosomes.^22,23^

Fluorescence-based probes offer a powerful solution for the quantitative detection of biologically relevant molecules at the subcellular level, owing to their high sensitivity, excellent temporal resolution, and capability of real-time monitoring in living cells.^24,25^ To fully realize these advantages, probes must be designed to possess specific organelle-targeting capacities, allowing for precise spatial analysis. In addition, incorporating ratiometric fluorescence signals with built-in self-calibration ensures more reliable and accurate quantification.^26^ In this context, we developed a lysosome-targeted ratiometric fluorescent nanoprobe (HR-MP) capable of real-time monitoring of dynamic ATP fluctuations within lysosomes during autophagy. HR-MP functions via ATP-induced Förster resonance energy transfer (FRET), enabling selective and quantitative visualization of ATP fluctuations in live cells. HR-MP provides a valuable tool for elucidating the potential regulatory roles of ATP in autophagy, advancing our understanding of its involvement in both cellular homeostasis and pathological conditions.

## 2 Experimental Section

### 2.1 Reagents and Instruments

Pluronic F-127, triethoxy­(3-isocyanatopropyl)­silane, Lipoamido-dPEG 8-acid (CPCDs),1,1,2,3,4,5-hexaphenyl-1*H*-silole (HPS), (3-aminopropyl)­triethoxysilane (APTES), 3-methyladenine (3-MA), 1-ethyl-3-(3-dimethyl aminopropyl) carbodiimide (EDC), *N*-hydroxysuccinimide (NHS), apyrase, carbonyl cyanide *m*-chlorophenyl hydrazone (CCCP), phosphate-buffered saline (PBS) tablet, ATP, ADP, AMP, CTP, UTP, TTP, GTP, anti-LC3 antibody, Hank’s Balanced Salt Solution (HBSS), Dulbecco’s modified Eagle’s media (DMEM), trypsin/EDTA solution, Fetal Bovine Serum (FBS) are obtained from Sigma-Aldrich. Two commercial trackers, LysoTracker Deep Red (LDR) and MitoTracker Deep Red (MDR) are obtained from Thermo-Fisher. 3-Morpholin-4-yl-propionic acid hydrochloride (MP) is obtained from J&K Scientific (Beijing, China).

Electrospray ionization mass spectrometry (ESI-MS) spectra are acquired on a Waters SQ Detector. Proton nuclear magnetic resonance (^1^HNMR, 500 MHz) and carbon nuclear magnetic resonance (^13^CNMR, 125 MHz) are performed on a Varian 500 MHz spectrometer. Transmission electron microscopy (TEM) images are acquired through a JEOL JEM-1400 Flash transmission electron microscope. Fluorescent emission spectra are evaluated on a Fluoromax-4 spectrophotometer. Ultraviolet–visible (UV–vis) spectra are acquired on a UV-2450 spectrophotometer. MTT analyses are performed on a TECAN infinite M1000 Pro (microplate reader). ζ-Potential experiments and dynamic light scattering (DLS) measurements are studied on a Malvern Zetasizer Nano ZS90 size analyzer. Confocal fluorescence images are obtained on an inverted Zeiss LSM800.

### 2.2 Synthesis

#### 2.2.1 Synthesis of RDT

RDM-DEA (Scheme S1, Supporting Information) is synthesized following previously reported literature.^27^ To a solution of RDM-DEA (160 mg, 0.30 mmol) in dry THF (20 mL) was slowly added to a solution of APTES (80 mg, 0.39 mmol) in dry THF for 30 min under a nitrogen atmosphere in an ice–water bath. After the reaction was completed, the solvent was evaporated, and the residue was purified by silica gel chromatography using ethyl acetate/dichloromethane (v/v, 15:1) to give RDT. ^1^H NMR (500 MHz, CDCl_3_): δ 7.86 (m, 1H, Ar–H), 7.47 (m, 2H, Ar–H), 7.09 (m, 1H, Ar–H), 6.43–6.26 (m, 6H, Ar–H), 3.83–3.70 (m, 6H, CH_2_), 3.37–3.30 (m, 14H, CH_2_), 3.16–3.10 (m, 3H, CH_2_), 2.67 (t, *J* = 5.0, 2H, CH_2_), 1.67 (t, *J* = 5.0 Hz, 2H, CH_2_), 1.63–1.54 (m, 3H, CH_2_), 1.23–1.14 (m, 21H, CH_3_), 0.65–0.59 (m, 2H, CH_2_). ^13^C NMR (125 MHz, CDCl_3_): δ 169.47, 159.10, 153.62, 153.41, 149.08, 132.94, 130.75, 128.67, 128.35, 124.02, 122.90, 108.38, 104.90, 97.83, 65.64, 58.49, 48.92, 47.77, 43.03, 39.42, 39.22, 23.77, 18.41, 12.71, 7.77. MS (ESI-MS): Calcd for C_42_H_62_N_6_O_6_Si [M + H^+^]: 776.09. Found 776.64.

#### 2.2.2 Synthesis of HR-MP

HPS (0.25 mg) and F127 (100 mg) were mixed and dissolved in dry THF (1.5 mL) in a glass vial (3.0 mL) and stirred for 1 h at room temperature to obtain a homogeneous solution. After evaporating the THF solvent with a gentle nitrogen flow, residual THF remaining in the film was removed under vacuum, and then redispersed in hydrochloride solution (0.85 M, 1.5 mL) with ultrasonication. 160 μL of TEOS was added dropwise and the solution was stirred for 2 h at room temperature before adding 40 μL RDT (4.0 mg, in DCM) and 70 μL APTES. The termination of the condensation should last 24 h at room temperature with stirring. Finally, the solution was filtered through a 0.22 μm syringe filter to remove large aggregates, followed by dialysis using a membrane with a molecular weight cutoff (MWCO) of 10,000 Da against Milli-Q water for 2 days to remove unreacted low-molecular-weight components. EDC (70 μL, 1.0 mg/mL) and NHS (70 μL, 1.0 mg/mL) were then added to the solution, stirring in the dark for 30 min before adding MP (160 μL, 1.0 mg/mL) and CPCDs (40 μL, 10 mg/mL) into the solution. The mixture was then stirred at room temperature for 24 h. Finally, the mixture was purified by following the same methods as those used above.

### 2.3 Quantum Yield Measurement

The quantum yield of HPS-loaded nanoparticles was calculated by comparison with coumarin 153 (*R* = 0.53 in ethanol) as a reference^27^ using eq 1

1 $$\Phi_{x}=\frac{F_{x}\cdot A_{\mathrm{st}}}{F_{\mathrm{st}}\cdot A_{x}}{\left(\frac{n_{x}}{n_{\mathrm{st}}}\right)}^{2}\Phi_{\mathrm{st}}$$

where *F* is the integrated area under the fluorescence spectra, *A* is the absorbance, *n* is the refractive index of the solvent, and Φ is the quantum yield. The index *x* denotes the sample and st denotes the standard of coumarin 153.

### 2.4 Cell Studies

#### 2.4.1 Cell Culture and Fluorescent Imaging

A total of 2 × 10^5^ MCF-7 cells were seeded on a glass-bottom microwell dish and incubated with 1 mL of DMEM supplemented with 10% (v/v) FBS and 1% (v/v) penicillin-streptomycin solution, then stained with HR-MP (1.0 mg/mL) for 1 h, washed with PBS, and then incubated with LDR (1.0 μM) or MDR (0.5 μM) for 10 min. Finally, living cell microscopy was then conducted immediately by Zeiss LSM800 using a 60× oil immersion objective lens.

#### 2.4.2 Detecting Lysosomal ATP Changes

MCF-7 cells were seeded on a glass-bottom dish following the protocol above. The next day, cells were treated with 1.0 mg/mL HR-MP for 1 h, after being rinsed with PBS, and were continuously treated with apyrase (1.0 U/mL) and CaCl_2_ (10 mM) to stimuli lysosomal ATP changes, respectively.

#### 2.4.3 Starvation Experiments

MCF-7 cells were first incubated with HR-MP (1.0 mg/mL) for 1 h, followed by washing with PBS. The cells were then incubated in HBSS for different time periods to induce autophagy. Subsequently, cells were stained with MDR (0.2 μM) for 15 min, washed, and subjected to fluorescence microscopy analysis.

#### 2.4.4 Inhibition of Autophagy with 3-MA

MCF-7 cells were initially incubated with HR-MP (1.0 mg/mL) for 1 h. Then, the cells were washed with PBS and incubated in HBSS with 3-MA (100 μM) at the same time slots. Living cell microscopy was conducted under a Zeiss LSM 800 microscopy.

#### 2.4.5 Cytotoxicity Assay

MCF-7 cells in 96-well plates were rinsed with PBS and treated with a culture medium supplemented with HR-MP at the desired concentration for 24 h. After that, the cytotoxicity of HR-MP was examined by standard MTT assay according to previous reports.^28^

#### 2.4.6 Cell Viability Assay of MCF-7 Cells in Starvation Conditions

MCF-7 cells in 96-well plates were rinsed with PBS and treated with a culture medium containing 10% (v/v) FBS and 1% (v/v) penicillin-streptomycin solution. Cells were treated with HR-MP (1.0 mg/mL) for 1 h, and then the cells were washed with PBS and incubated with HBSS at the desired time. After that, the cytotoxicity of HR-MP was examined by a standard MTT assay.

#### 2.4.7 Western Blot

Western blot experiments were based on our previously reported work.^29^

## 3 Results and Discussion

### 3.1 Fabrication of the Ratiometric ATP Nanoprobe (HR-MP)

The fabrication of HR-MP employed a strategy like our previous work on lysosomal pH sensors.^28,29^ The primary difference lies in the design of the responsive unit RDT, which is tailored to respond to ATP through the hydrogen bond-induced ring-opening of rhodamine B spirolactam.^30^ As depicted in Figure 1a,b, RDT is a rhodamine B moiety suspended with diethylenetriamine (DEA), which can open the spirolactam ring *via* specific multiple hydrogen bonds between the amino groups of DEA and the phosphate groups of ATP, as well as π-stacking interactions between the rhodamine core and adenine of ATP.^31–33^ The resulting fluorescent xanthene can then serve as an efficient FRET acceptor in the constructed FRET nanosystem (Figure 1b). Details on the synthesis of RDT are provided in the Supporting Information (Figures S1–S3 and Scheme S1). Second, a highly hydrophobic AIEgen (high emissive aggregation-induced emission luminogen), hexaphenylsilole (HPS), is selected as the FRET donor due to its outstanding AIE properties and high quantum yield (0.57). In addition, the emission band of HPS (centered at 480 nm) overlaps well with the absorption band of xanthene (Figure 1c). HPS can be easily encapsulated into the hydrophobic core of the nanoplatform, forming micelles with significant AIE characteristics, and the encapsulated HPS aggregates exhibit insensitivity to common bioanalytes (Figure S4). Finally, lysosome-targeting HR-MP with the capability of specifically responding to ATP was achieved by silane-cross-linking of HPS, RDT, and a light-transparent (UV–vis) triblock copolymer of F-127, followed by further surface modifications with morpholine (MP) for lysosomal targeting and cell-penetrating cyclic disulfide (CPCDs) for enhanced cellular penetration (Figure 1a). The morphology of HR-MP was characterized by transmission electron microscopy (TEM), displaying a uniform spherical core–shell structure (Figure 1d and the inset). The size distribution obtained by dynamic light scattering (DLS) indicates an average diameter of approximately 29 nm (Figure 2a), slightly larger than those sizes observed in the TEM image of dried structures. This discrepancy likely arises from the influence of the dispersed surface ligands in aqueous environments on DLS measurements.^34,35^

**Figure 1:**
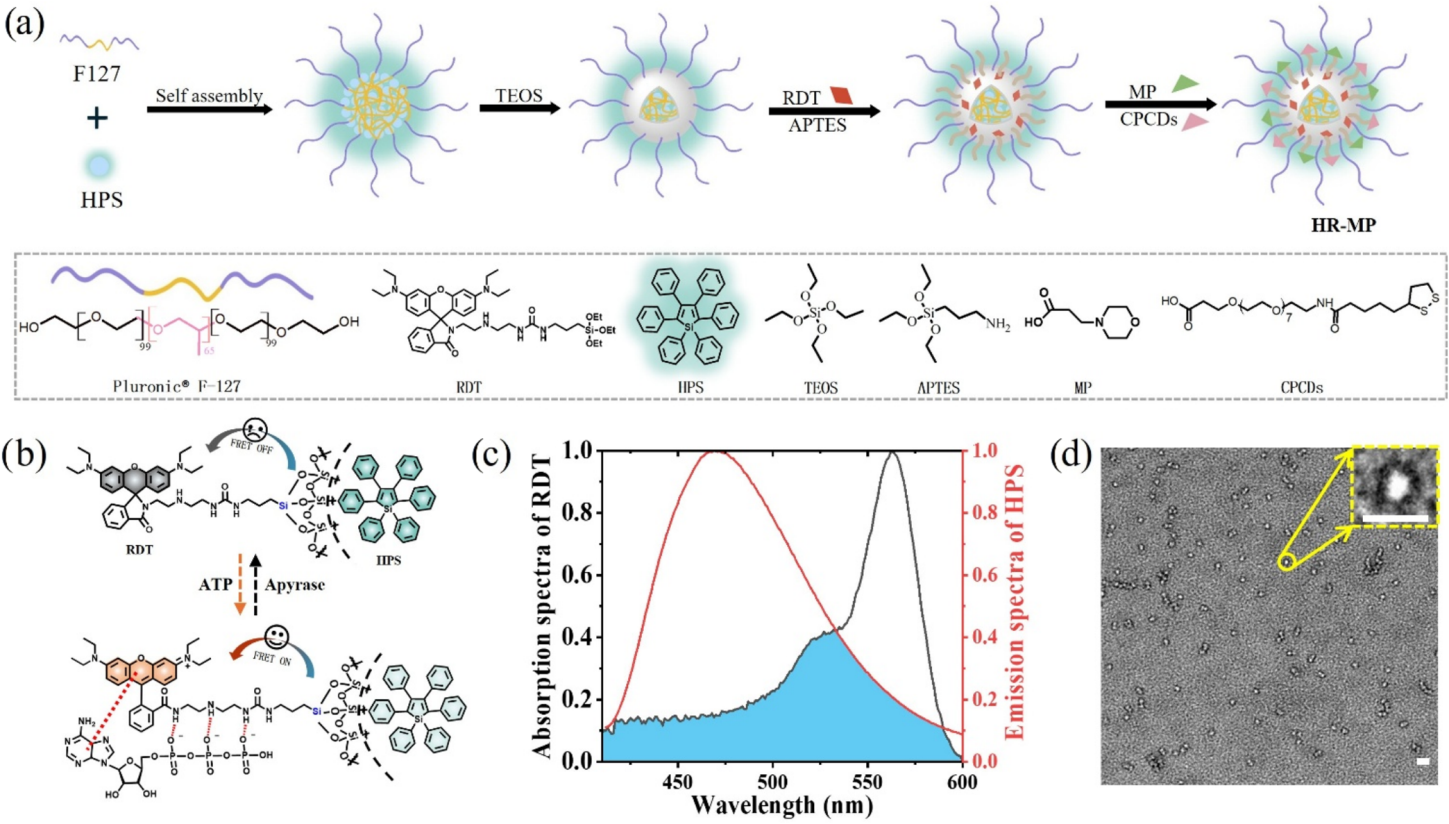
(a) Schematic illustration of the preparation of HR-MP. (b) Schematic switchable FRET of HR-MP upon ATP response. (c) Normalized fluorescence of HPS-loaded NPs (red line) and absorption of RDT-loaded NPs saturated with ATP (black line). (d) Transmission electron microscopy (TEM) image of HR-MP, scale bar = 20 nm.

**Figure 2:**
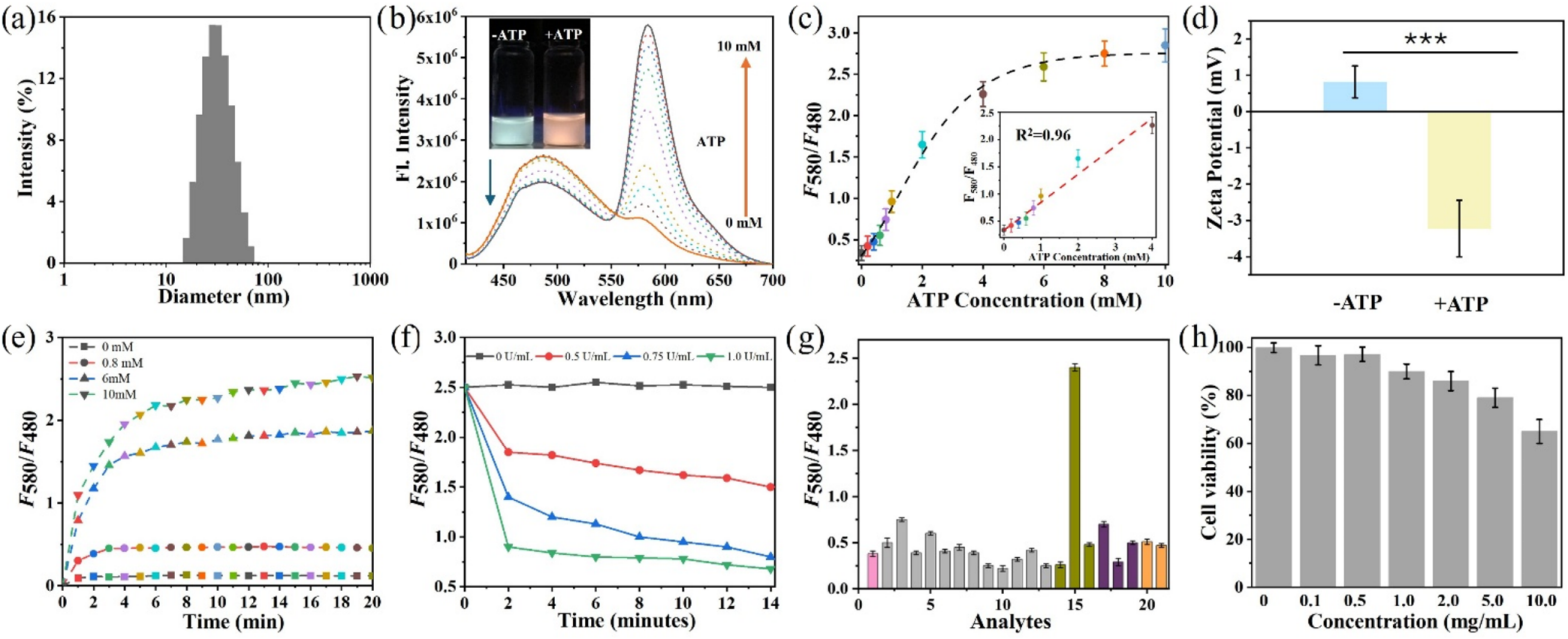
(a) Average size distribution of HR-MP by dynamic light scattering (DLS). (b) Fluorescence emission spectra of HR-MP (1.0 mg/mL) in buffer solutions with ATP changes under 400 nm excitation. Inset: photographs of HR-MP with (right) and without (left) ATP in buffer at pH 5.0 under 365 nm irradiation. (c) Fluorescent ratio changes of HR-MP responding to increasing concentrations of ATP. (d) Zeta-potential changes of HR-MP before and after ATP binding in aqueous solutions. (e) Time-dependent fluorescence intensity ratio (*F*_580_/*F*_480_) of HR-MP in response to varying ATP concentrations at pH 5.0. (f) Time-dependent fluorescence ratio (*F*_580_/*F*_480_) changes of HR-MP saturated with ATP upon addition of apyrase (0–1.0 U/mL) (pH 5.0). (g) Fluorescence response of HR-MP toward various analytes (**1**: probe (1.0 mg/mL); **2–8**: K^+^, Na^+^, Zn^2+^, Cu^2+^, Mg^2+^, Ba^2+^, and Ca^2+^ (10 μM); **9–11**: H_2_PO_4_^–^, HPO_4_^2–^, and PO_4_^3–^ (10 μM); **12–14**: GSH, Hcy, and Cys (10 μM); **15–21**: ATP, ADP, AMP, CTP, UTP, TTP, GTP (10 mM)). (h) Cell viability assay of MCF-7 cells after incubation with different concentrations of HR-MP for 24 h.

### 3.2 Spectral Response of HR-MP to ATP *In Vitro*

To verify that HR-MP can monitor ATP levels in the lysosomal environment, the spectral response of HR-MP was studied to an increasing concentration of ATP in a weak-acidic buffer solution (pH 5.0, PBS 0.01 M). As shown in Figure 2b, under excitation of HPS (λ_ex_ = 400 nm), HR-MP exhibits a strong blue emission of HPS. Upon addition of ATP, a significant increase in the emission intensity at 580 nm and a slight decrease at 480 nm are observed, resulting in a clear ratiometric signal change between the dual emission signals. This response is attributed to the “ring-opening” of the spirolactam and the subsequent activation of xanthene fluorescence in RDT upon ATP binding, which then facilitates a FRET process from HPS to the xanthene unit (Figure 1b). An ATP calibration curve was subsequently established (Figure 2c), demonstrating a linear relationship between the ratiometric signal and ATP concentration in the range of 0–4.0 mM (*R* ^2^ = 0.96). The limit of detection (LOD) was calculated to be 16.7 μM using the formula LOD = 3*σ/S. Furthermore, to test the influence of pH, the fluorescence intensity of HR-MP with ATP (concentration from 0 to 10 mM) was measured over a pH range of 4.0–8.0. As shown in Figure S5, HR-MP responds to ATP more efficiently in the acidic lysosomal pH range rather than in the cytosolic and mitochondrial pH ranges, ensuring its reliability of the ATP response in lysosomes. Meanwhile, the addition of ATP results in a significant decrease in the ζ-potential of HR-MP (Figure 2d) from 0.814 ± 0.44 to −3.23 ± 0.78 mV, with a slight increase in size of HR-MP from 29.1 to 31 nm (Figure S6), which was caused by the binding between HR-MP and ATP.^36^

Further, the time kinetic experiments of HR-MP toward ATP at different concentrations (0, 0.8, 6, and 10 mM) were studied to evaluate the possibility of dynamically monitoring intracellular ATP levels. As shown in Figure 2e, upon addition of various concentrations of ATP to the buffer solution (pH = 5) of HR-MP, the ratio of fluorescence intensity at 480 and 580 nm (*F*_580_/*F*_480_) increases sharply and reaches equilibrium within 5 min, showing that HR-MP can respond to ATP rapidly. In the absence of ATP, no obvious change in the fluorescence ratio (*F*_580_/*F*_480_) is observed. These results show that HR-MP is well-suited for the dynamic detection of ATP changes. The reversibility of the HR-MP and ATP interaction was further evaluated using apyrase, an enzyme that catalyzes the hydrolysis of ATP into AMP.^37^ Upon addition of different concentrations of apyrase to the acidic HR-MP solution in the presence of 10 mM ATP, the fluorescence ratios (*F*_580_/*F*_480_) of the solution decrease with increasing amounts of apyrase (Figure 2f), suggesting that the recognition process is reversible. Therefore, HR-MP enables real-time monitoring of ATP fluctuations.

To examine the specific response of HR-MP toward ATP, HR-MP was treated with a variety of analytes, including metals, ions, and various biosubstances, followed by the measurement of their fluorescence spectra and calculation of fluorescence ratio (*F*_580_/*F*_480_). As shown in Figure 2g, HR-MP exhibits a specific response to ATP, with no significant changes in fluorescence ratio upon addition of other competitive analytes. Due to their structural similarities, ADP or UTP often interferes with ATP detection when using fluorescent probes.^32,38,39^ Notably, our results demonstrate that HR-MP exhibits negligible responses toward ADP, UTP, or other nucleoside polyphosphates (NPPs, structure shown in Figure S7). The remarkable selectivity is attributed not only to the π–π interactions between xanthene and adenine, as well as multiple hydrogen bonds between amino and phosphate groups,^24^ but also to the surface effects of the nanoplatform. These surface effects promote a favorable molecular orientation of RDT, facilitating the desired interactions.

### 3.3 Ratiometric Studies of HR-MP to ATP in Living Cells

To explore the application of HR-MP imaging in some biological progress, the MTT assay was first employed to evaluate its cytotoxicity. As shown in Figure 2h, HR-MP shows negligible cytotoxicity in MCF-7 cells, with an excellent cell viability of >90% at a concentration of 1 mg/mL after incubation for 24 h, indicating that 1 mg/mL of HR-MP can be safely used in subsequent cell imaging experiments.

To evaluate the lysosomal targeting capability of HR-MP, MCF-7 cells were coincubated with HR-MP (1 mg/mL) and LysoTracker Deep Red (LDR), a commercially available lysosomal marker. Fluorescence signals in the green and red channels correspond to unbound and ATP-bound HR-MP, respectively, while the LDR channel highlights lysosomes. As illustrated in Figure 3a, both green and red fluorescence exhibited a granular pattern with an average size of approximately 0.89 μm (Figure S8), indicating efficient lysosomal staining. Moreover, the red fluorescent puncta from HR-MP overlapped significantly with those of LDR-stained regions, as evidenced by a strong Pearson correlation coefficient (*R*_r_ = 0.89) (Figure 3a). The line profiling of the pixel intensity of interest also suggests that the signals from HR-MP closely matched the LDR staining (Figure 3b). Control experiments using LDR alone showed no detectable fluorescence in either the green or red channels, confirming that the observed signals originated exclusively from HR-MP (Figure S9a). It is worth noting that mitochondria, known as the cellular energy factories, are the primary sites of intracellular ATP generation.^40^ So, an additional colocalization experiment was conducted to further investigate whether mitochondrial ATP affects the sensing capability of HR-MP. The poor spatial overlap between HR-MP and MitoTracker Deep Red (MDR) was observed, indicating that HR-MP does not respond to mitochondrial ATP and further demonstrating its high lysosome-specificity (Figure S9b). This further demonstrates the efficient lysosome-targeting ability of this nanoplatform and highlights the unique ATP-sensing properties of HR-MP. Overall, the results demonstrate that HR-MP fulfills the design requirement for specific lysosomal localization, thereby ensuring reliable monitoring of lysosomal ATP levels.

**Figure 3:**
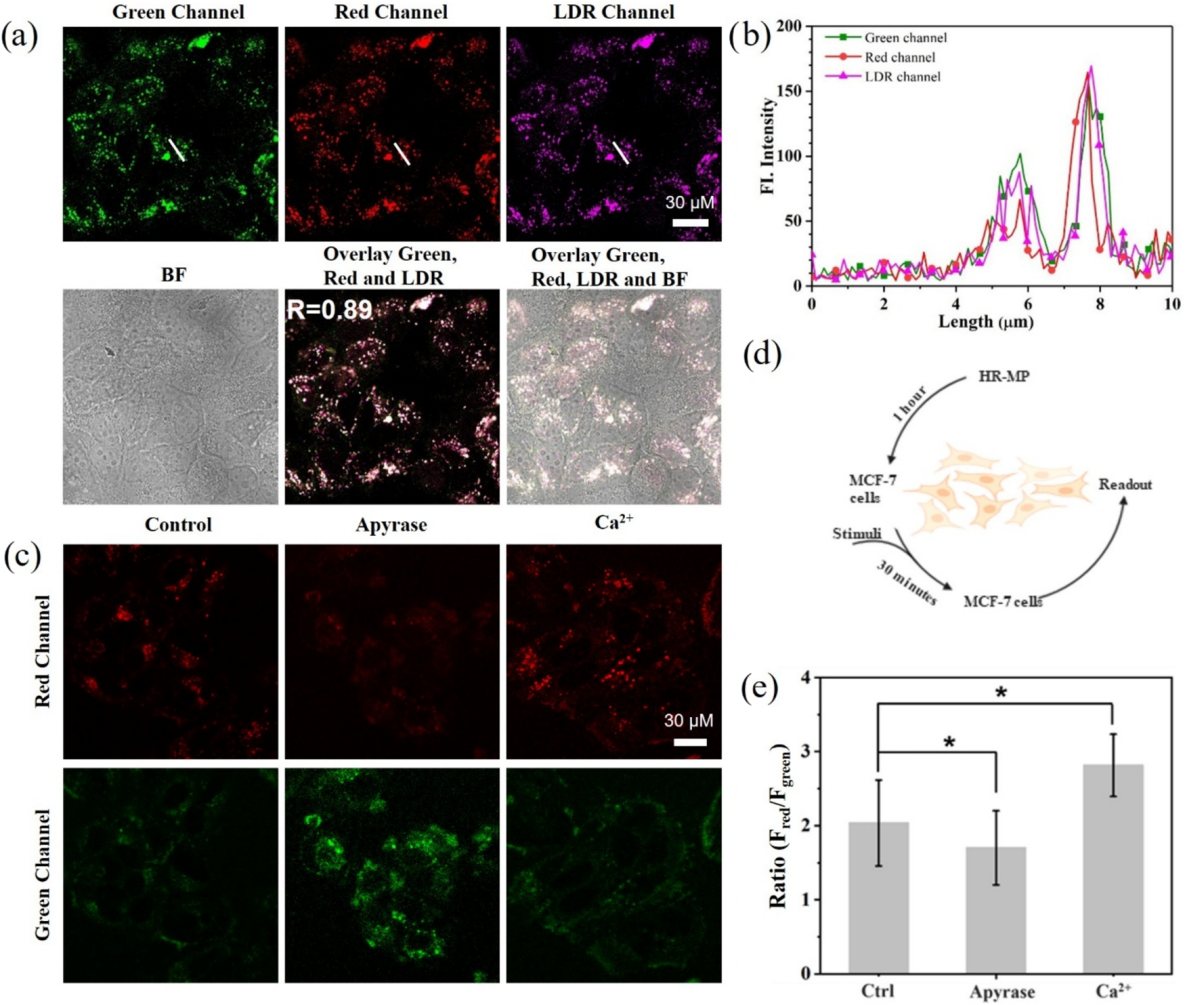
(a) Fluorescent images of MCF-7 cells costained with HR-MP (1.0 mg/mL) and LDR (1.0 μM). (b) Fluorescent intensity profile of regions of interest (ROI) in panel (a). (c) Fluorescent images of MCF-7 cells preincubated with HR-MP (1.0 mg/mL) for 1 h and then treated with apyrase (1.0 U/mL) and Ca^2+^ (10 mM), respectively. (d) Schematic workflow of stimulus induction experimental procedures. (e) Summarized data of stimuli-induced lysosomal ATP changes. Red channel: Ex, 405 nm, Em, 560–600 nm; Green channel: Ex, 405 nm, Em, 460–500 nm; LDR channel: Ex, 647 nm, Em, 658–668 nm.

Given the abundance of ATP within lysosomes, abnormal fluctuations in lysosomal ATP levels can significantly impact cellular functions. Therefore, quantitative detection of the lysosomal ATP concentration in the cells is essential. First, to verify the capability of HR-MP for quantitative detection of ATP under microscopy, the fluorescence imaging was performed on glass slides with drop-cast HR-MP samples with varying ATP concentrations at pH 5.0. The results confirmed a reliable and concentration-dependent ratiometric fluorescence response (Figure S10). Next, to verify the reliability of HR-MP’s ratiometric signal for ATP-sensing performance in the intracellular environment, MCF-7 cells were treated with exogenous ATP (3 mM) at pH 5.0 in the presence of nigericin, a proton ionophore, which can homogenize intracellular and extracellular pH rapidly.^41,42^ The cells were then incubated with varying concentrations of HR-MP, and the ratios of *F*_red_/*F*_green_ were measured (Figure S11). The results demonstrated that the fluorescence ratio remained nearly constant across different concentrations of HR-MP, indicating the ratiometric response of HR-MP to ATP is independent of the concentration of HR-MP. To establish an intracellular calibration curve, 1 mg/mL HR-MP was used to detect exogenous ATP with MCF-7 cells at pH 5.0 (Figure S12). The resulting fluorescence ratios increased linearly with ATP concentration, consistent with the quantitative detection behavior observed in the drop-cast experiments (Figure S11). These findings demonstrate that HR-MP can be reliably used for the quantitative determination of intracellular ATP.

Therefore, monitoring changes in lysosomal ATP in living cells is crucial for gaining insights into lysosome-associated autophagic processes. To validate the sensing capability of HR-MP, MCF-7 cells were incubated with the probe and subsequently exposed to various stimuli, including apyrase and Ca^2+^, to modulate cellular ATP levels.^36,43^ As shown in Figure 3c,e, treatment with adenosine apyrase (1.0 U/mL), a highly active ATP-hydrolyzing enzyme, led to a decrease in fluorescence intensity in the red channel, indicating reduced ATP levels following enzymatic hydrolysis. More importantly, HR-MP was also capable of real-time tracking of ATP elevation upon nutrient supplementation in the culture medium (Figure S13). In contrast, treatment of MCF-7 cells with Ca^2+^ (10 mM), a known stimulator of ATP production, significantly increased lysosomal ATP levels, as evidenced by an elevated fluorescence ratio (*F*_red_/*F*_green_, Figure 3e).^44^ In summary, these results remarkably demonstrate the capability and advantages of HR-MP for real-time monitoring of lysosomal ATP dynamics.

### 3.4 Autophagy Studies to Track ATP in Living Cells

Energy metabolism is one fundamental characteristic of life activities. The lysosome-mediated autophagy process is regulated by energy metabolism and, in turn, plays a role in regulating energy metabolism. Therefore, investigating the relationship between energy metabolism and cellular autophagy is of practical significance for the pathological study of certain diseases.^45^ To study their correlation, we first established an autophagy model using Hank’s Balanced Salt Solution (HBSS), a nutrient-free medium commonly used to induce nonspecific autophagy.^46^ As shown in Figures 4a (red channel and green channel) and b, the cells were incubated in HBSS for 3 and 6 h, and the fluorescence intensity in the red channel decreased, while the fluorescence in the green channel increased, leading to a decrease in the ratio of the two channels (*F*_red_/*F*_green_) . It shows a significant reduction of lysosomal ATP concentration from 4.12 to 0.97 mM, following 6 h of glucose starvation. However, when the starvation period was extended to 9 h, ATP concentration slightly increased to 2.23 mM (Figure 4b). Additionally, ATP continued to increase slowly and reached 2.92 mM under 24 h of starvation. Further cell viability experiments were conducted to determine whether this increase in ATP levels upon starvation from 3 to 24 h was due to general cell lysis.^47^ After 24 h of starvation, MCF-7 cells maintained a high cell viability of higher than 80% (Figure 4c), negating the possibility of general cell lysis. In addition, the starvation environment with incubation of an autophagy inhibitor, the antimalarial agent 3-MA,^48^ results in significant decreases in ATP levels within 24 h (Figure S14). It suggests that the reduction in ATP levels within lysosomes during the early stages of starvation is due to the energy required to maintain normal cell function and the energy consumed by autophagy.^11,49^ As shown in Figure S15, under 3 h of starvation, the expression of LC3-II was significantly increased, showing that the autophagy was triggered. As starvation continues, the increase in ATP levels might be attributed to mitophagy. Along with mitophagy progress, dysfunctional mitochondria containing substantial amounts of ATP would be engulfed by lysosomes for degradation, resulting in an obvious increase in lysosomal ATP levels.

**Figure 4:**
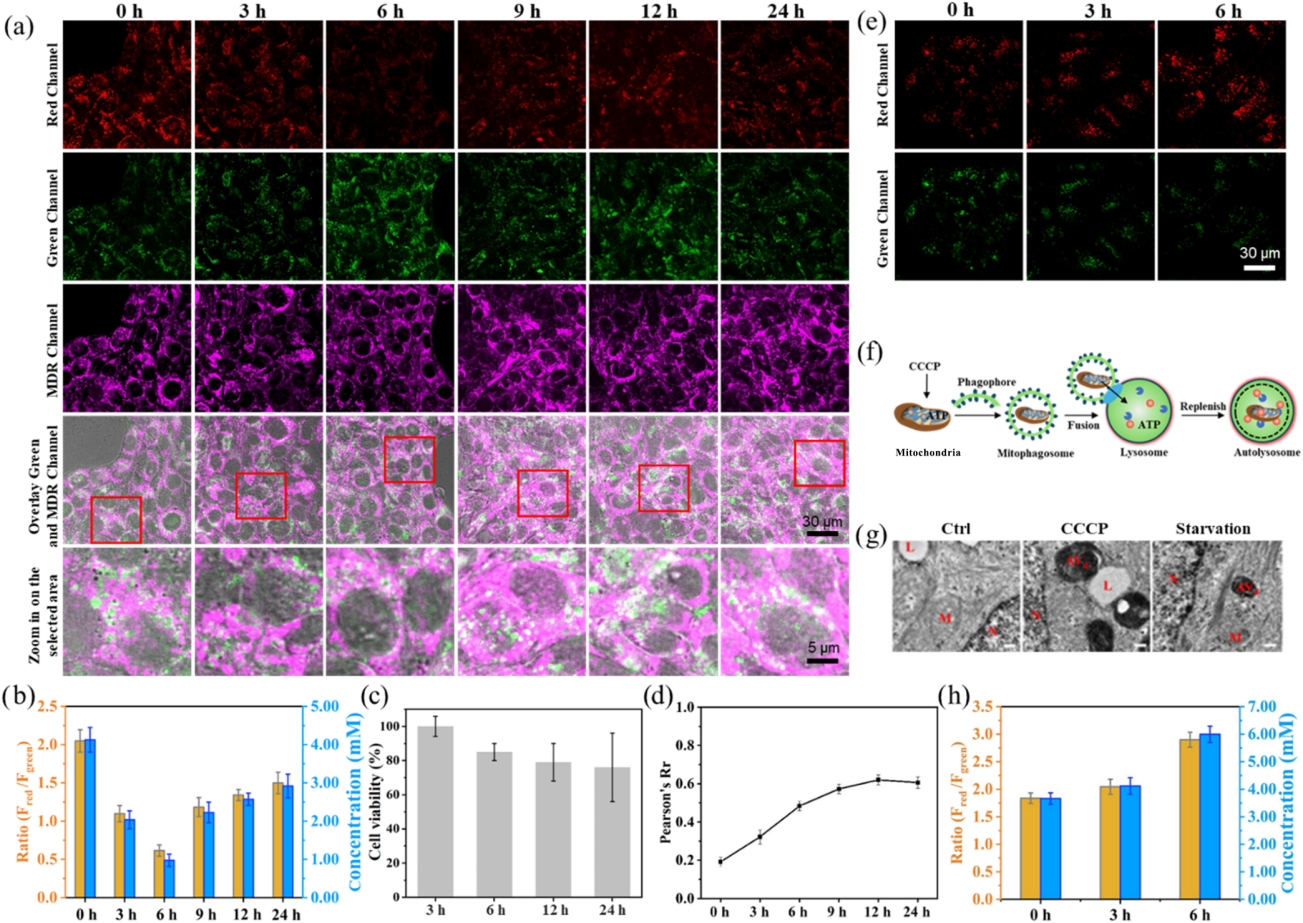
(a) Fluorescent images of autophagy (scale bar: 30 and 5 μm) and (b) fluorescence ratios (*F*_red_/*F*_green_) and lysosomal ATP concentrations of MCF-7 cells prestained with HR-MP (1.0 mg/mL) in HBSS medium with costained with MDR (0.2 μM). (c) Cell viability assay of MCF-7 cells with diverse starvation times. (d) Degree of fusion between mitochondria and lysosomes over the time of starvation. (e) Fluorescent images of mitophagy (scale bar: 30 μm) in MCF-7 cells with HR-MP (1.0 mg/mL) in the presence of CCCP (10 μM) incubation. (f) Schematic illustration of CCCP-induced mitophagy process. (g) TEM cell images with different treatments; scale bar: 500 nm. N: nucleus, L: lysosome, M: mitochondrion, AV_d_: degradative autophagic vacuole. (h) Fluorescence ratios (*F*_red_/*F*_green_) and lysosomal ATP concentrations in MCF-7 cells with HR-MP (1.0 mg/mL) in the presence of CCCP (10 μM). Red channel: Ex, 405 nm; Em, 560–600 nm; Green channel: Ex, 405 nm, Em, 460–500 nm. MDR channel: Ex, 633 nm, Em, 650–670 nm.

To validate the rationality of the above hypothesis, we simulated two types of mitophagy processes. One is nonselective mitophagy induced by starvation stress, a commonly observed process reported in previous studies.^50–52^ The other is selective mitophagy induced by carbonyl cyanide *m*-chlorophenyl hydrazone (CCCP, a membrane potential uncoupler that can induce mitophagy).^53^ As shown in Figure 4a (MDR channel) and 4d, after 3 and 6 h of starvation, the extent of mitochondrial-lysosomal fusion increased from 30 to 50%. It shows that the nonselective mitophagy is initiated as early as 3 h of starvation and is enhanced with continued starvation up to 9 h. After the subsequent 12 h of starvation, mitophagy reaches a steady state and experiences a slight weakening. As mentioned above, the autophagy process is energy-consuming. During the early stages of starvation, cellular energy consumption exceeds the ATP carried by the damaged mitochondria that fuse with lysosomes, which leads to a remarkable decrease in ATP (Figure 4b,d). In the later stages of starvation, the fusion between damaged mitochondria and lysosomes slightly decreases, while ATP levels increase, indicating that lysosomes have degraded the damaged mitochondria or other materials to produce energy.

Since CCCP-induced mitophagy is a selective degradation of excess and dysfunctional mitochondria in the lysosomes,^54^ it is crucial to utilize the ratiometric-sensing capability of HR-MP to further track ATP changes in lysosomal ATP during mitophagy for studying the complex autophagy process. Specifically, MCF-7 cells were incubated with HR-MP for 1 h; CCCP was then added to depolarize the mitochondrial membrane (Figure 4e–g). As can be seen from Figure 4h, the fluorescence ratio of CCCP-treated MCF-7 cells gradually increased over time, which is unlikely to be caused by pH, since mitophagy typically leads to an increase in lysosomal pH.^55^ Thus, this strongly suggests that ATP levels within lysosomes significantly increase following CCCP treatment. This should be attributed to the fusion of mitochondria with lysosomes, resulting in the release of ATP from the damaged mitochondria into the lysosomes. The fluorescence ratio shows a much more significant increase under 6 h of CCCP treatment, and the ATP level increases to 6.0 mM, which is significantly higher than that of cells under 24 h of starvation (2.92 mM). It indicates that mitophagy is a more effective process to increase lysosomal ATP levels compared to starvation-induced nonspecific autophagy. In addition, Western Blot (WB) experiments were performed to confirm the occurrence of autophagy. As presented in Figures S15 and S16, the expression of LC3-II was significantly increased with time under starvation and CCCP treatment, leading to a rise in the ratio of LC3-II/LC3-I, which indicates the activation of autophagy. Overall, the results demonstrate that the occurrence of autophagy is critical for the maintenance of lysosomal ATP levels, while mitophagy was shown to be more effective in increasing lysosomal ATP concentrations.

## 4 Conclusions

In conclusion, autophagy is essential for cell survival under metabolic stress and provides substrates for energy production under such stress. Leveraging its high precision and quantitative capabilities, our ratiometric nanoprobe HR-MP was employed to quantitatively monitor lysosomal ATP variations under various stimulation conditions. Additionally, the association between autophagy and energy metabolism in lysosomes was demonstrated for the first time using a fluorescent nanoprobe. These findings confirm that autophagy is an energy-consuming process and plays a critical role in maintaining lysosomal ATP homeostasis. Compared to starvation-induced nonselective autophagy, mitophagy is more effective in enhancing lysosomal ATP levels. This novel probe not only advances our understanding of how autophagy contributes to cellular functions but also opens new avenues for investigating the mechanisms underlying autophagy and its regulation of energy metabolism. The application of HR-MP could lead to significant insights into diseases characterized by impaired autophagic flux or energy imbalance, potentially guiding the development of therapeutic strategies targeting autophagy-related pathways

## Supplementary Material



## Notes

^1^H NMR, ^13^C NMR, and ESI-MS data of compounds; pH investigation and DLS data of HR-MP; structures of the nucleoside polyphosphates; quantification analysis of ATP in MCF-7 cells and Western blot data (PDF)
