# Supplementary material for: Real-Time Monitoring of Adenosine Triphosphate Fluctuation in Lysosome during Autophagy/Mitophagy
Source: ACS Appl Mater Interfaces. 2025 Jul 11;17(29):41719–28. doi: 10.1021/acsami.5c07496 (PMC12291090; doi:10.1021/acsami.5c07496)
Supplement: Supplementary file 1 [file am5c07496_si_001.pdf]

Supporting Information

Real-time Monitoring of Adenosine Triphosphate Fluctuation in Lysosome during Autophagy/Mitophagy

Jiwen Hu [a,‡], Hong Wang [b,‡], Xin Zhang [a,‡], Chunfei Wang [c], Anna du Rietz [a], Mengtao Rong [b], Caroline Brommesson [a], Xiongyu Wu [a], Zhanxiao Wei [a], Ruilong Zhang [b], Xuanjun Zhang [c], Kajsa Uvdal [a], Zhangjun Hu [a,*]

[a] Department of Physics, Chemistry, and Biology (IFM), Linköping University, Linköping SE-581 83, Sweden

[b] School of Chemistry and Chemical Engineering, and Institute of Physical Science and Information Technology, Anhui University, Hefei, Anhui 230601, China

[c] Cancer Centre and Centre of Reproduction, Development and Aging, Faculty of Health Science, University of Macau, Taipa, Macau SAR 999078 (China)

[‡] These authors contributed equally to this work.

E-mail: zhangjun.hu@liu.se

Scheme and Figures

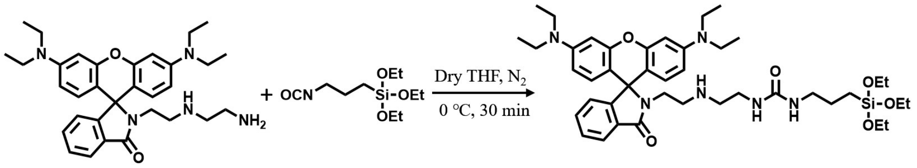

RDM-DEA   Triethoxy(3-isocyanatopropyl)silane   RDT

Scheme S1. Synthesis of RDT.

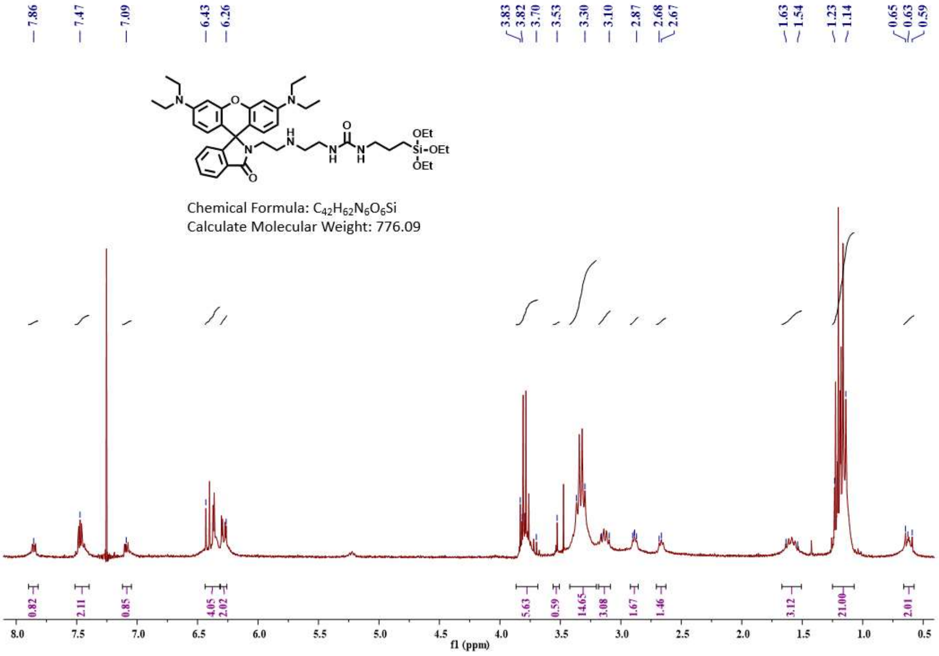

Figure S1. ^{1}H-NMR spectrum of RDT in $CDCl_3$.

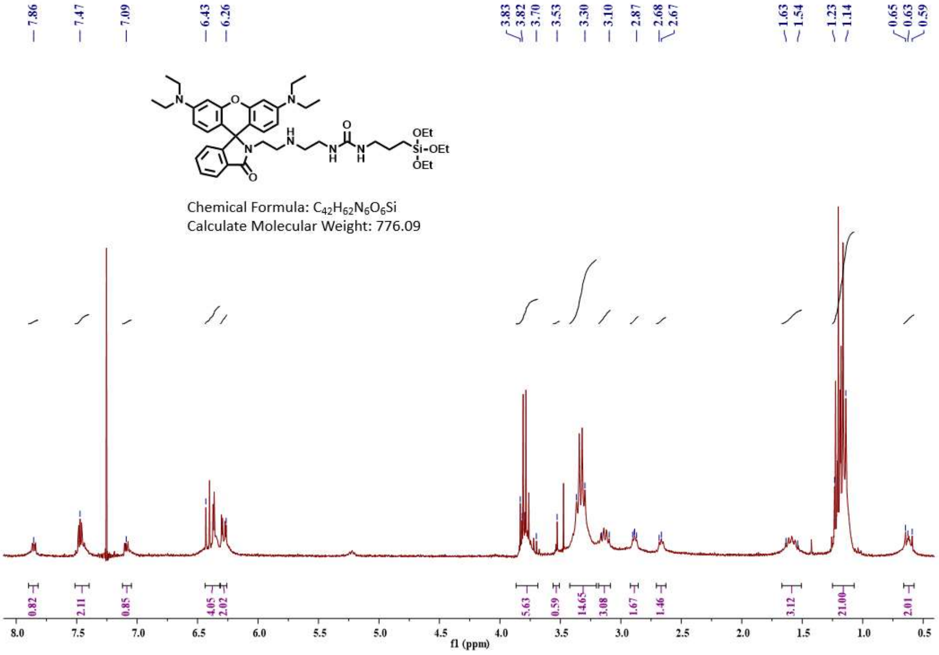

Figure S2. ^{13}C-NMR spectrum of RDT in $CDCl_3$.

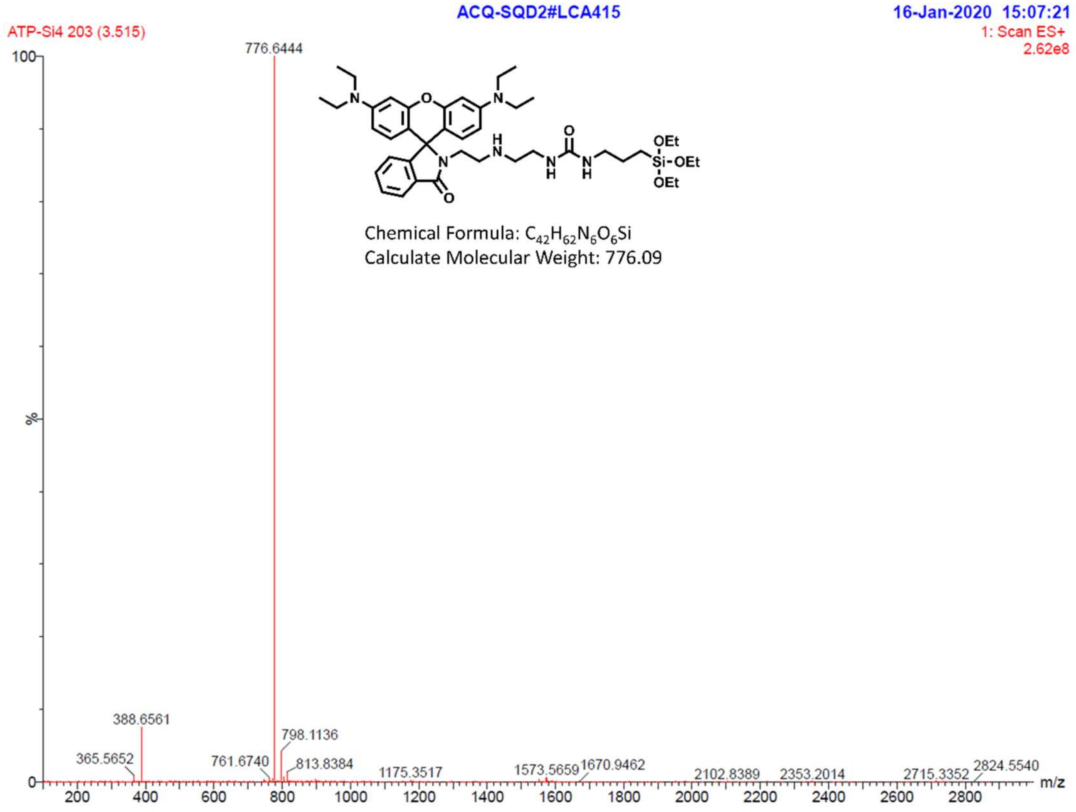

Figure S3. ESI-MS spectrum of RDT.

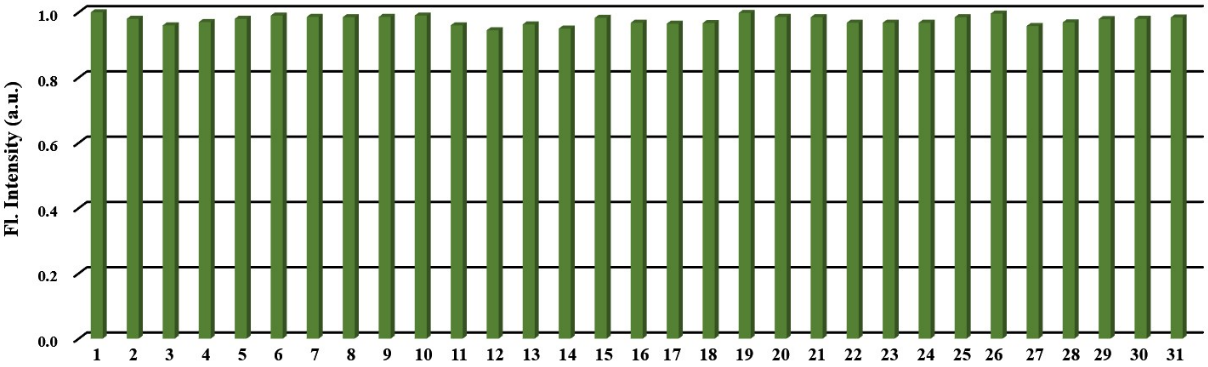

Figure S4. Fluorescence response of HPS (1.0 μM) toward various analytes (1: blank; cations (2-16): K^+, Ni^+, Ag^+, Na^+, Mn^{2+}, Pb^{2+}, Cd^{2+}, Fe^{2+}, Zn^{2+}, Cu^{2+}, Mg^{2+}, Ba^{2+}, Ca^{2+}, Co^{2+}, Cr^{3+}; anions (17-24): OH^-, SO_4^{2-}, NO_3^-, CO_3^{2-}, Cl^-, $H_2PO_4^-$, HPO_4^{2-}, PO_4^{3-}; NPPs (25-31): ATP, ADP, AMP, CTP, UTP, TTP, GTP (10 mM).

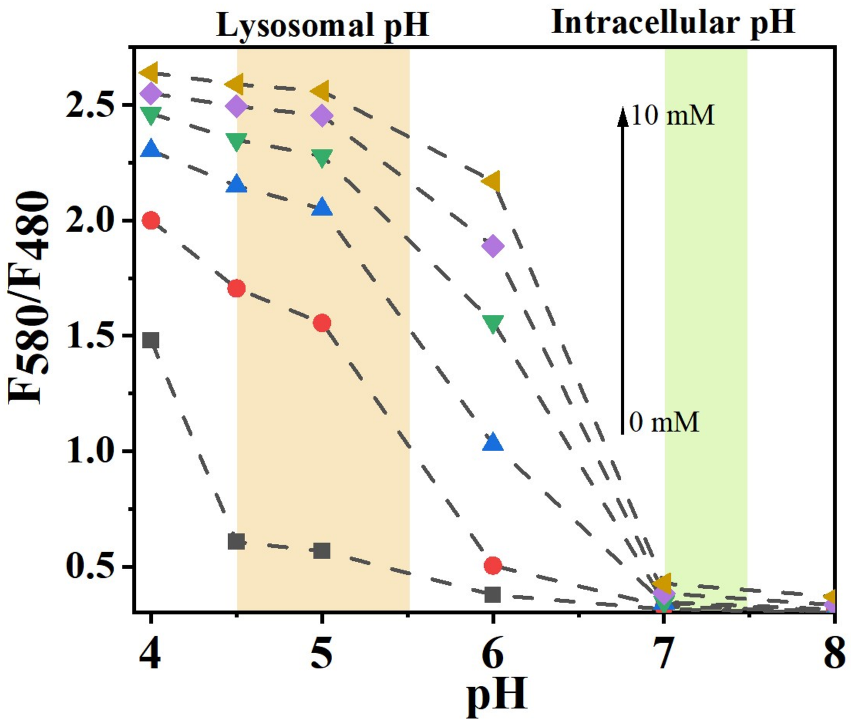

Figure S5. pH dependency of HR-MP for the detection of ATP.

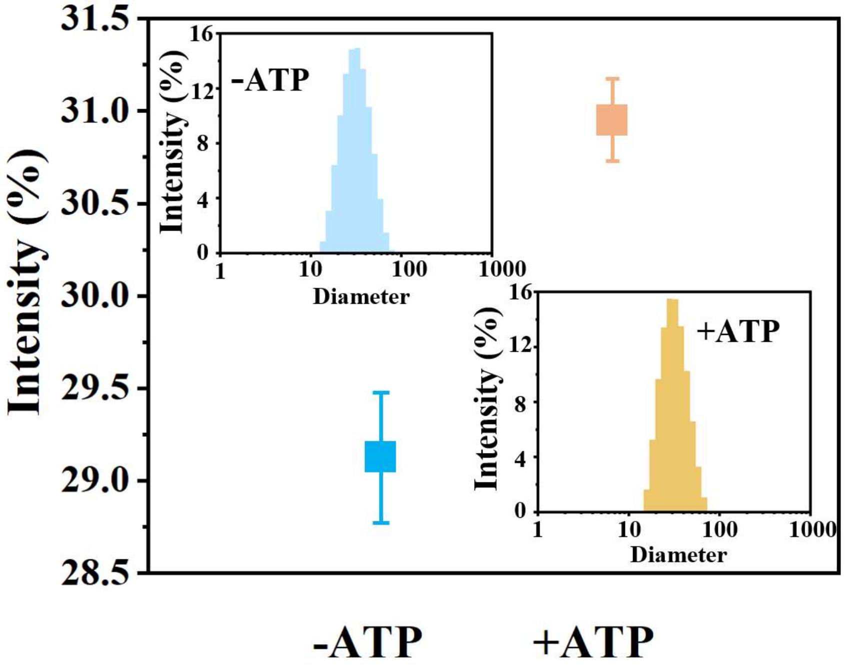

Figure S6. Summarized data of ATP-induced size changes of HR-MP determined by DLS. Inset: size distribution diagram of HR-MP with and without ATP treatment.

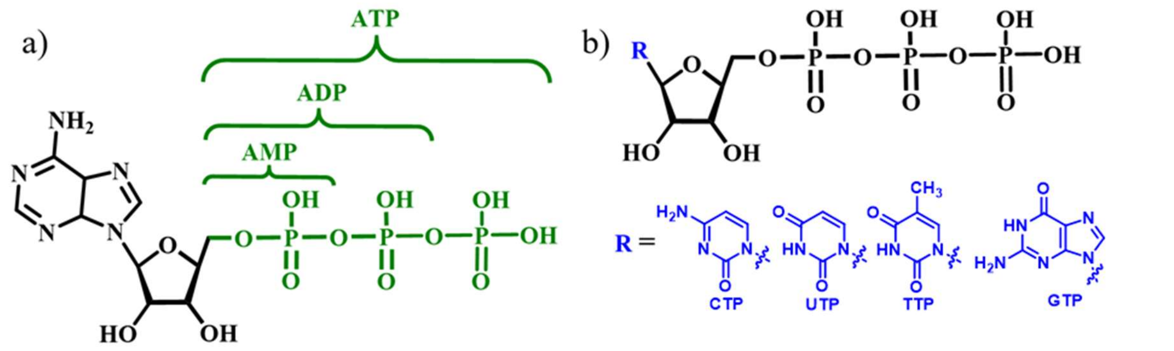

Figure S7. Schematic structure of nucleoside polyphosphates (NPPS). (a) AMP, ADP, ATP. (b) CTP, UTP, TTP.

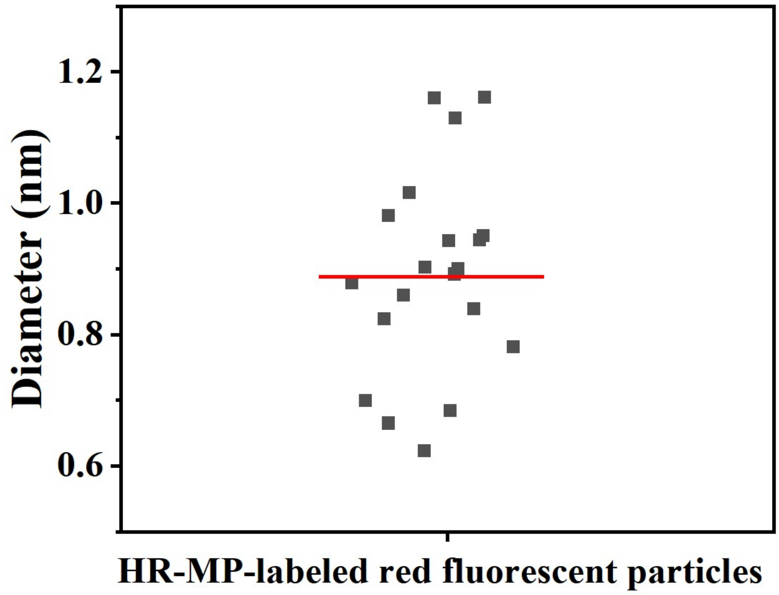

Figure S8. Diameters of HR-MP-labelled fluorescent particles in red channel of MCF-7 cells.

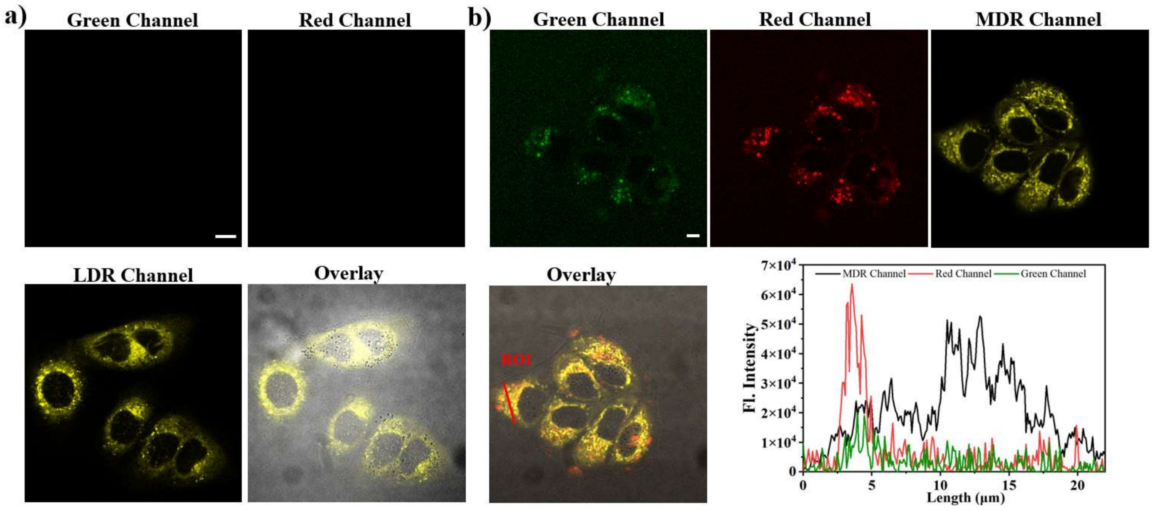

Figure S9. (a) Fluorescent images of MCF-7 cells after 10 min of incubation with LDR (1.0 μM). (b) Fluorescent images of MCF-7 cells co-stained with HR-MP (1.0 mg/mL) and MDR (1.0 μM), and fluorescent intensity profile of regions of interest (ROI) in panel b (red line in overlay). Red channel: Ex, 405 nm, Em, 560-600 nm; Green channel: Ex, 405 nm, Em, 460-500 nm; LDR channel: Ex, 647 nm, Em, 658-668 nm, scale bar = 10 μm.

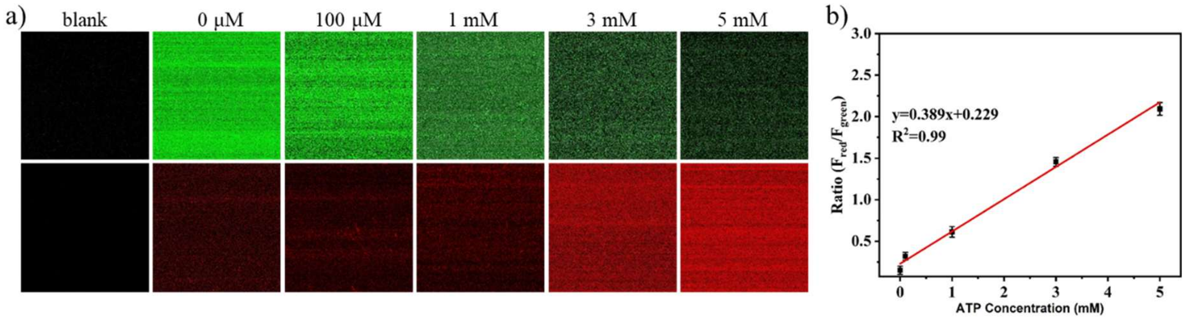

Figure S10. (a) Fluorescent images and (b) fluorescence intensities ratios (F_{red}/F_{green}) of 1 mg/mL HR-MP solution dropped on a glass slide under varying ATP concentrations at pH 5.0. Red channel: Ex, 405 nm, Em, 560-600 nm; Green channel: Ex, 405 nm, Em, 460-500 nm.

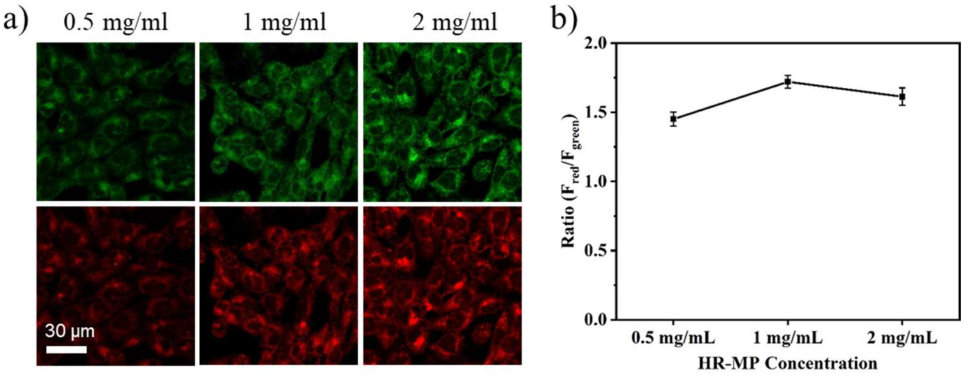

Figure S11. (a) Fluorescent images and (b) fluorescence intensities ratios (F_{red}/F_{green}) of MCF-7 cells pre-treated with nigericin (10 μM) and then incubated with varying concentration of HR-MP (0.5 mg/mL, 1 mg/mL, 2 mg/mL) under fixed concentration of ATP (3 mM). Red channel: Ex, 405 nm, Em, 560-600 nm; Green channel: Ex, 405 nm, Em, 460-500 nm. Scale bar = 30 μm.

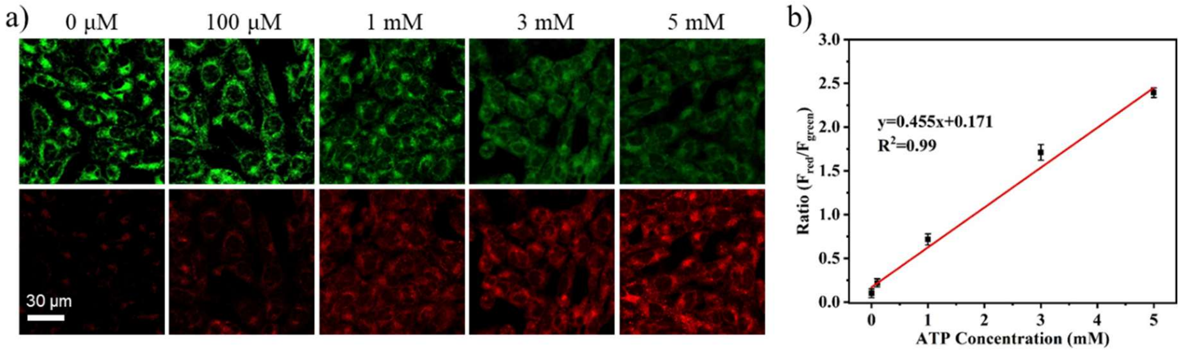

Figure S12. (a) Fluorescent images and (b) fluorescence intensities ratios (F_{red}/F_{green}) of MCF-7 cells pre-treated with nigericin (10 μM) and then incubated with HR-MP (1 mg/mL) under varying exogenous ATP concentrations (0-5 mM) at pH 5.0. Red channel: Ex, 405 nm, Em, 560-600 nm; Green channel: Ex, 405 nm, Em, 460-500 nm. Scale bar = 30 μm.

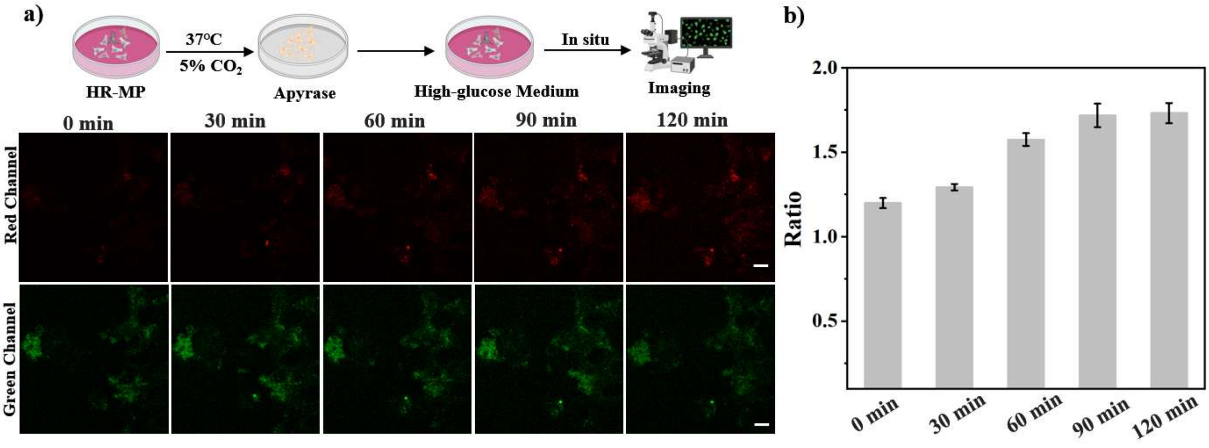

Figure S13. (a) Schematic workflow and real-time fluorescent images of MCF-7 cells after the addition of high glucose medium containing HR-MP (1.0 mg/mL). (b) Summarized data of lysosomal ATP changes after the addition of high glucose medium. Red channel: Ex, 405 nm, Em, 560-600 nm; Green channel: Ex, 405 nm, Em, 460-500 nm; LDR channel: Ex, 647 nm, Em, 658-668 nm, scale bar = 10 µm.

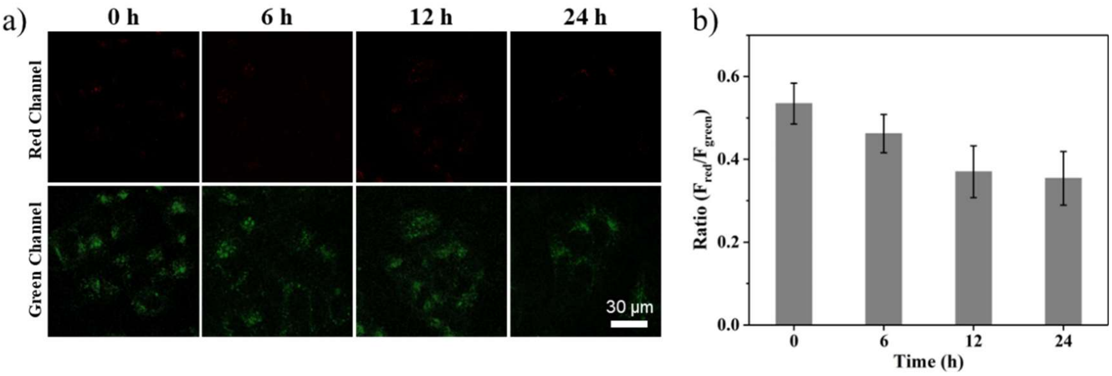

Figure S14. (a) Fluorescent images and (b) fluorescence ratios (F_{red}/F_{green}) of MCF-7 cells pre-stained with 1.0 mg/mL of HR-MP in HBSS with 3-MA (100 µM) treatment. Red channel: Ex, 405 nm, Em, 560-600 nm; Green channel: Ex, 405 nm, Em, 460-500 nm, scale bar = 30 µm.

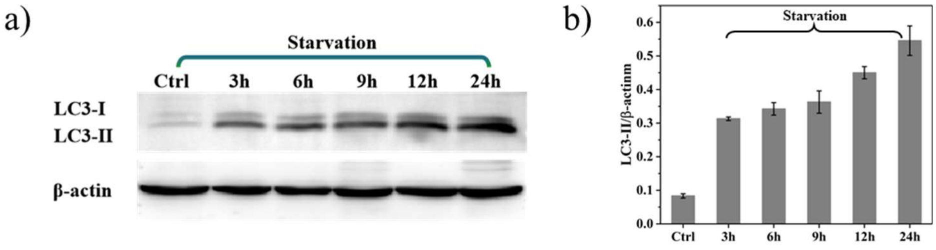

Figure S15. (a) Autophagy-related protein expression and (b) statistical analysis of LC3-II under starvation at different hours.

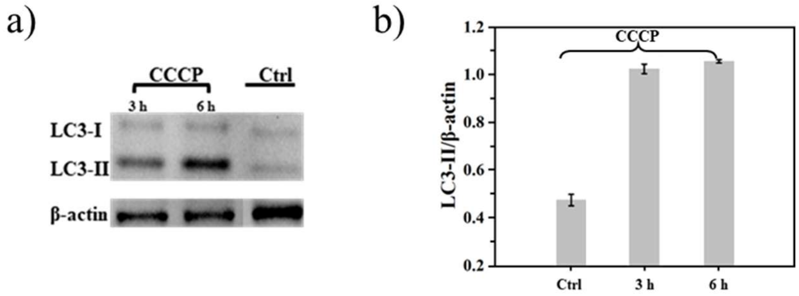

Figure S16. (a) Autophagy-related protein expression and (b) statistical analysis of LC3-II under treatment of CCCP (10 μM) at different hours.
